# Effects of cooking methods on total isothiocyanate yield from cruciferous vegetables

**DOI:** 10.1002/fsn3.1836

**Published:** 2020-09-09

**Authors:** Zinian Wang, Marilyn L. Kwan, Rachel Pratt, Janise M. Roh, Lawrence H. Kushi, Kim N. Danforth, Yuesheng Zhang, Christine B. Ambrosone, Li Tang

**Affiliations:** ^1^ Department of Cancer Prevention and Control Roswell Park Comprehensive Cancer Center Buffalo NY USA; ^2^ Division of Research Kaiser Permanente Northern California Oakland CA USA; ^3^ Department of Research and Evaluation Kaiser Permanente Southern California Pasadena CA USA; ^4^ Department of Pharmacology and Therapeutics Roswell Park Comprehensive Cancer Center Buffalo NY USA

**Keywords:** cancer chemoprevention, cooking methods, cruciferous vegetable, dietary exposure, isothiocyanate yield, phytochemicals

## Abstract

Cruciferous vegetables are primary sources of dietary isothiocyanates (ITCs), a group of phytochemicals showing promising cancer‐chemopreventive activities in multiple cancer models. However, no study has thoroughly examined how cooking affects the yields of ITCs from cruciferous vegetables. In this study, a high‐performance liquid chromatography (HPLC)‐based cyclocondensation assay was performed to examine the ITC yields from four major cruciferous vegetables (broccoli, cabbage, cauliflower, and kale) under six cooking conditions (stir‐frying, steaming, microwaving, boiling, stewing, and chip‐baking for kale only) and measured the level of ITCs under the raw condition for a comprehensive list of cruciferous vegetables and ITC‐containing condiments. A wide range of ITC yields was found across vegetables and condiments. Cooking significantly altered the ITC yields, showing an averagely four‐fold increase by lightly cooking (stir‐frying, steaming, and microwaving) and a 58% decrease by heavily cooking (boiling, stewing, and chip‐baking). These findings will provide the evidence‐based cooking guidance on cruciferous vegetable consumption and help better estimate dietary ITC exposure in epidemiologic studies.

## Graphical abstract

Cooking significantly altered the isothiocyanate yields, showing an averagely four‐fold increase by lightly cooking (stir‐frying, steaming, and microwaving) and a 58% decrease by heavily cooking (boiling, stewing, and chip‐baking).

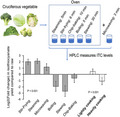

## 1 INTRODUCTION

The cancer‐preventive potential of cruciferous vegetables has drawn public and research interests, primarily due to their unique phytochemicals, dietary isothiocyanates (ITCs). Cruciferous vegetables consist of a diverse group of vegetables containing glucosinolates, the precursors of ITCs (Holst & Williamson, 2004). Glucosinolates are segregated from the endogenous enzyme myrosinase in intact plants. When vegetables are chopped or chewed, glucosinolates are hydrolyzed due to the released myrosinase (Higdon, Delage, Williams, & Dashwood, 2007). ITCs are one of the hydrolyzed products from glucosinolates and have long been known to be biologically active because of its chemical structure of –N=C=S group (Fenwick, Heaney, Mullin, & VanEtten, 1983). The anticancer property of dietary ITCs, which was not recognized until the early 1990s, has been supported by a rapid growth of preclinical evidence in various cancer models (Chung, Morse, Eklind, & Lewis, 1992; Singh & Singh, 2012; Tang, Paonessa, Zhang, Ambrosone, & McCann, 2013). Multi‐faceted anticancer mechanisms have been identified for dietary ITCs (Mokhtari et al., 2018; Royston & Tollefsbol, 2015). For example, dietary ITCs have been shown to inhibit phase I carcinogen‐activating enzymes (Guo et al., 1992) and to induce phase II carcinogen‐detoxifying enzymes (Sparnins, Venegas, & Wattenberg, 1982), thus blocking carcinogenesis and preventing cancer initiation and/or progression (Hecht, 1999).

With strong evidence from preclinical studies, there is a growing interest in estimation of dietary ITC exposure in humans to understand its role in cancer chemoprevention. However, two reasons hindered progress in the field. First, food composition data for dietary ITCs were not available until recently, and only for a limited list of cruciferous vegetables (Jiao, Yu, Hankin, Low, & Chung, 1998; Tang et al., 2013). Second, although several studies have shown that cooking reduces the amount of glucosinolates or ITCs obtained from vegetables (Kapusta‐Duch, Kusznierewicz, Leszczyńska, & Borczak, 2016; Rouzaud, Young, & Duncan, 2004), many questions remain unanswered, including whether different cooking methods have varied impact on the ITC yield, to what extent cooking affects the ITC yield, and whether the effect of cooking differs by type of vegetables. Some studies tested the impact of cooking methods on glucosinolate levels, but the results were not consistent. For example, Song and Thornalley (2007) found that boiling significantly decreased levels of glucosinolates while steaming, microwaving, and stir‐frying did not. In contrast, Jones, Frisina, Winkler, Imsic, and Tomkins (2010) found that both boiling and microwaving significantly decreased glucosinolate levels.

It is important to note that total glucosinolate content in cruciferous vegetables does not reflect total ITC yield. Several studies have documented glucosinolate contents in cruciferous vegetables and reported as many as 15 different glucosinolates in a single vegetable (Agudo et al., 2008; Kushad et al., 1999; McNaughton & Marks, 2003; Verkerk et al., 2009). Depending on the chemical structure of glucosinolates such as aliphatic, aromatic, or indole glucosinolates, plant‐intrinsic factors including myrosinase, epithiospecifier protein (ESP), ascorbic acid, and Fe^2+^, as well as extrinsic factors such as pH, temperature, and pressure, glucosinolates can be hydrolyzed to release various end products, including ITCs, indoles, nitriles, and thiocyanates (Burow, Markert, Gershenzon, & Wittstock, 2006; Fahey, Zalcmann, & Talalay, 2001; Oliviero, Verkerk, & Dekker, 2018). Of all factors, myrosinase and ESP play critical but opposite roles in production of ITCs from glucosinolates. Myrosinase initiates the hydrolysis of glucosinolates, resulting in formation of unstable intermediates, which rearrange to form ITCs; while ESP interacts with the unstable intermediates to divert ITC formation into nitriles, which has not shown any anti‐cancer potential. Interestingly, the substrate specificity of ESP varies by chemical structure of glucosinolates, showing a high efficiency on the hydrolysis of aliphatic glucosinolates compared with aromatic glucosinolates (Cole, 1978; Kaoulla, MacLeod, & Gil, 1980; Matusheski, Juvik, & Jeffery, 2004; Matusheski et al., 2006; Petroski & Tookey, 1982; Wittstock & Burow, 2007). Also the presence of ESP varies in vegetables with a strong activity in broccoli but not in mustard or horseradish (Cole, 1978; Kaoulla et al., 1980; Matusheski et al., 2004, 2006; Petroski & Tookey, 1982; Wittstock & Burow, 2007). Therefore, the ITC yield could differ considerably in vegetables even if they contain similar type and/or similar amount of glucosinolates.

In addition, the hydrolysis of glucosinolates could occur in the human gastrointestinal tract by myrosinase‐containing microflora (Getahun & Chung, 1999; Shapiro, Fahey, Wade, Stephenson, & Talalay, 1998). However, the efficiency and amount of ITCs generated by gastrointestinal microflora could be relatively low and vary substantially by individuals. Clinical trials involving healthy volunteers reported 2%–50% recovery rates of administered glucoraphanin (the precursor of sulforaphane, one of the widely studied ITCs in broccoli) as ITC metabolites in urine samples among individuals (Enger et al., 2011; Kensler et al., 2005; Shapiro et al., 2006). An average 18% recovery rate with a dose of 100 µmol glucoraphanin (Shapiro et al., 2006) and an average 5% recovery rate with a dose of 800 µmol glucoraphanin were reported (Enger et al., 2011). On the contrary, our previous study found that hydrolysis of glucosinolates in the vegetables occurs fast and efficiently, and the amount of myrosinase in cruciferous vegetables alone is sufficient for complete hydrolysis of glucosinolates under the raw condition (Tang et al., 2013). Therefore, direct measurement of ITC yields from cruciferous vegetables provides better estimates of ITC exposure from dietary intake.

In this study, we aimed to provide a comprehensive ITC food composition database and weighting factors for the estimation of dietary ITC exposure under different cooking methods. A total of 21 types of cruciferous vegetables and four types of condiments were analyzed under the raw condition. At least three samples from each of the four commonly consumed cruciferous vegetables in Western diets—broccoli, cabbage, cauliflower, and kale—were prepared under the conditions representing six cooking methods (stir‐frying, steaming, microwaving, boiling, stewing, and chip‐baking for kale only). A quantitative high‐performance liquid chromatography (HPLC)‐based assay was used to directly assess ITC yields from cruciferous vegetables and the effect of cooking methods on ITC yields.

## 2 MATERIALS AND METHODS

### 2.1 Materials

1,2‐Benzenedithiol was purchased from Sigma‐Aldrich, purified by vacuum distillation, and stored in small aliquots at −20°C. Sulforaphane, a major dietary ITC, was purchased from LKT Laboratories. HPLC grade Methanol was purchased from Fisher Chemical.

### 2.2 Sample collection and preparation

Based on the literature (Thomson et al., 2007), frequency of consumption, and the availability in the United States, a list of 21 commonly consumed cruciferous vegetables was assembled, including arugula, broccoli, broccolini, brussels sprouts, Chinese cabbage, green/white cabbage (shown as cabbage below), red/purple cabbage, cauliflower, bok choy (also known as Chinese white cabbage), yu choy, gai lan (also known as Chinese broccoli), collard green, kale, mustard green, turnip, rapini (also known as broccoli rabe), daikon, kohlrabi, radish, watercress, and sauerkraut. Four ITC‐containing condiments, including yellow mustard, mayonnaise, horseradish, and wasabi, were also included in the study. About 3–5 samples from each of the 21 types of cruciferous vegetables and four types of condiments were purchased from various supermarkets on different dates in Buffalo, New York, a metropolitan area in efforts to diversify the sources of cruciferous vegetables and the brands of the condiments. Each sample was weighted and homogenized with deionized water at 1 :3 ratio (weight:volume) in a Waring glass blender (VWR). Approximately 30 g of each fresh vegetable and 10 g of each condiment were used. After homogenization, the mixture was centrifuged at 640 xg for 5 min at 4°C to remove insoluble materials. The supernatant was stored at −80°C until HPLC analysis. All vegetables and condiments were processed on the day of purchase.

### 2.3 Preparation of cooked samples

Four commonly consumed cruciferous vegetables in Western diet—broccoli, cabbage, cauliflower, and kale—were chosen to evaluate the effect of cooking methods on the yields of ITCs. To standardize cooking procedures across the samples, approximately 30 g of each fresh vegetable was cooked in the laboratory using a Sheldon Manufacturing oven at same temperature (210°C) but with different volumes of deionized water and for different cooking times to mimic boiling, stewing, steaming, and stir‐frying, along with microwaving. The detailed cooking condition for each cooking method is listed in Table 1. Given the popularity of kale chips, chip‐baking was added for kale only. At least three samples were tested for each vegetable under each cooking method. After cooking, vegetables were removed from the cooking water, homogenized with fresh deionized water at the ratio of 1:3 (weight:volume) in a Waring glass blender (VWR), and processed as the raw counterparts.

**Table 1:** Cooking water and cooking time under different cooking methods for cruciferous vegetables

	Cooking methods	Weight (g)	Water volume (ml)	Cooking time (min)^a^
Lightly cooking	Steaming^b^	30	200	5
	Stir‐frying	30	0	6
	Microwaving	30	150	3
Heavily cooking	Boiling	30	150	5
	Stewing	30	150	30
	Chip‐baking (Kale only)	30	0	10

^a^ Cooking time was proportionately shortened giving 30 g of vegetables being cooked.

^b^ Vegetables were separated from water during steaming.

### 2.4 Cyclocondensation assay

**Figure 1:**
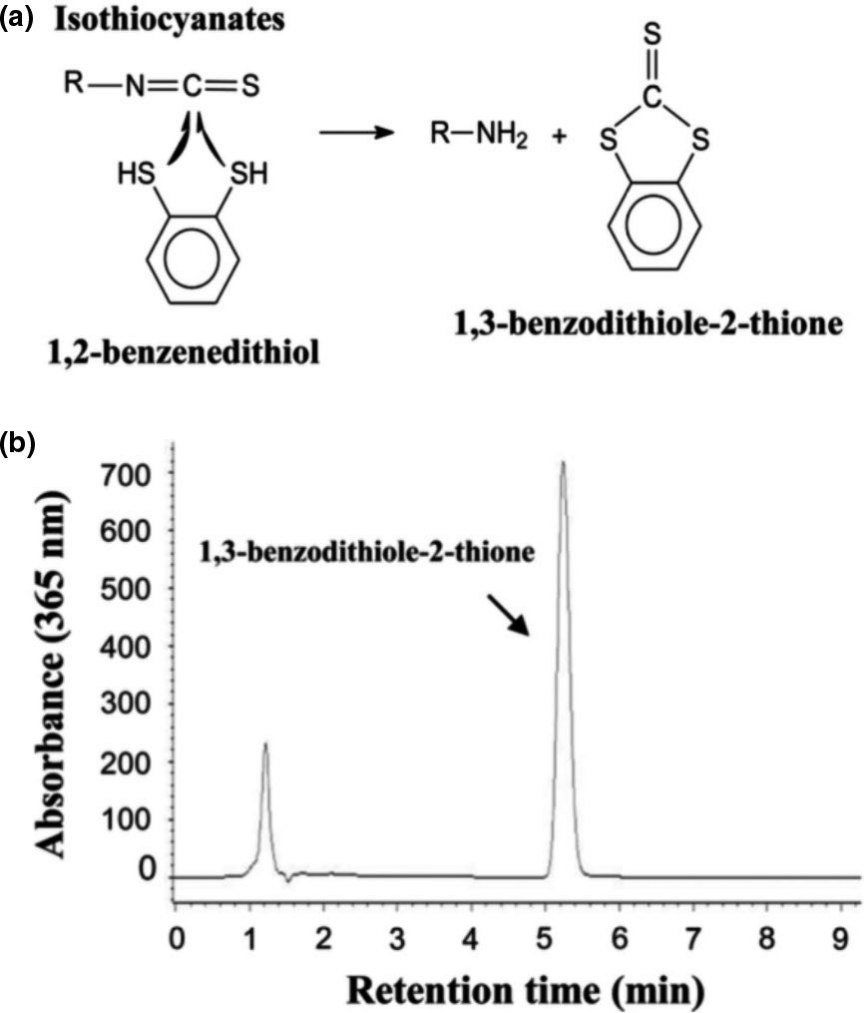
Chemical reaction of HPLC‐based cyclocondensation assay. (a) 1,3‐benzenedithiole‐2‐thione derived from reaction of isothiocyanates and 1,2‐benzenedithiol; (b) A typical HPLC chromatogram of 1,3‐benzenedithiole‐2‐thione (Adapted from Zhang, 2012)

ITC levels were measured using a previously validated method termed HPLC‐based cyclocondensation assay (Tang et al., 2013; Zhang, Wade, Prestera, & Talalay, 1996). The cyclocondensation assay relies on the reaction between the –N=C=S group of isothiocyanates and the thiol group of 1,2‐benzenedithiol to form a 1,3‐benzodithiole‐2‐thione and the corresponding amine (Figure 1a). Using a simple reverse‐phase HPLC, 1,3‐benzodithiole‐2‐thione is eluted at 5–6 min at 365 nm (Figure 1b). This method does not differentiate individual ITCs and only measures the total ITCs (Zhang, 2012). In a 1 ml reaction (adjusted with water), each sample (up to 100 µl) was mixed with 400 µl methanol, 250 µl of 100 mM potassium phosphate buffer (pH 8.5) and 100 µl of 1,2‐benzenedithiol at the concentration of 1.24 g/ml in methanol in a 4‐ml glass vial with a screw cap. Duplicate reactions were prepared for each sample. The reaction mixture was incubated at 65°C for 2 hr and centrifuged at low speed for 10 min. A total of 300 µl of supernatant were loaded into an autosampler and analyzed by HPLC. A blank control (no samples) and a series of standards (5, 10, and 20 µl of 500 µM sulforaphane) were included in each run. An Agilent HPLC system equipped with a model G1311B pump, a model G1329B autosampler, a model G1315C photodiode array detector, and an Agilent Chemostation chromatography data system was coupled to an analytical C18 reverse‐phase column (HiCHROM, Partisil 10 μm ODS‐2, 250 × 9.4 mm) for the cyclocondensation assay. The mobile phase consisted of methanol (80%) and H_2_O (20%) running at a flow rate of 1.75 ml/min with a sample injection volume of 100 μl and a detection wavelength at 365 nm. Coefficient of variation (CV, the ratio of the standard deviation to the mean) was used for quality control. A close value (CV < 10%) of total ITC levels was obtained for the duplicate samples, and their average was used for the analyses.

### 2.5 Statistical analysis

ITC yields were presented as means and ranges. To understand the cooking effect, fold changes were calculated for each vegetable using the ITC yield under each cooking method over the yield from the raw. Due to non‐normal distribution, the fold changes were presented as medians and interquartile ranges (IQR). One‐way ANOVA (analysis of variance) was used for comparison of fold changes after log2 transformation. A nonparametric Kruskal–Wallis test followed by Dunn post hoc test was performed for comparison of each cooking method to the raw (Keppel & Wickens, 2004). A *p*‐value less than .05 was considered statistically significant. All analyses were performed in RStudio (version 1.0.153).

## 3 RESULTS

### 3.1 ITC yields from raw cruciferous vegetables and condiments

A comprehensive list of ITC‐containing foods was assembled, including 21 types of commonly consumed cruciferous vegetables and four types of condiments. The ITC yields were measured under the uncooked condition to represent the ITC exposure after raw consumption. The means and ranges of total ITC yields from these raw vegetables and condiments are shown in Table 2. Cruciferous vegetables had a wide range of ITC yields (as much as 300‐fold difference). On average, collard greens had the lowest yield of ITCs (0.7 μmol/100 g wet weight), while arugula had the highest level (206.9 μmol/100 g wet weight). The largest intravariation in ITC yields was observed among three samples of kale, ranging from 0.2 to 10.3 μmol/100 g wet weight with a 51‐fold difference. In contrast, bok choy had relatively consistent ITC yields with an average 4.3–5.6 μmol/100 g wet weight across three types of bok choy (bok choy, white baby bok choy, and green baby bok choy). Turnip greens and roots were analyzed separately given that both commonly appear in human diet. Turnip roots had over seven‐fold higher yield of ITCs compared with greens (31.4 vs. 4.3 μmol/100 g wet weight), indicating a disproportionate distribution of glucosinolates in parts of vegetables. Among the four condiments, ITC levels were highest in horseradish (295.1 μmol/100 g wet weight) and lowest in mayonnaise (2.8 μmol/100 g wet weight). Variations in ITC levels were also observed within each type of condiment. For example, horseradish had an average of 295.1 μmol/100 g wet weight, but ranged from 87.1 to 639.8 μmol/100 g wet weight across four brands of horseradish. Overall, ITC yields varied across all samples, as well as within each type of sample for both vegetables and condiments.

**Table 2:** Yield of isothiocyanates (ITCs) from uncooked cruciferous vegetables and ITC‐containing condiments

	*N*	Mean (ITC µmol/100 g)	Range
Arugula	3	206.9	74.2–304.1
Broccoli	4	5.7	2.7–11.7
Broccolini	3	39.0	3.3–100.5
Broccoli sprouts	3	21.0	13.5–34.0
Brussels sprouts	3	9.0	3.3–15.0
Cauliflower	4	2.3	1.3–2.9
Gai Lan (Chinese broccoli)	4	3.1	0.3–9.3
Rapini (broccoli rabe)	3	7.7	1.2–11.7
Watercress	4	61.6	23.4–93.2
Cabbage			
Chinese/Napa cabbage	3	2.2	1.5–3.1
Green/white cabbage	4	57.6	40.2–67.1
Red/purple cabbage	3	2.8	1.3–3.8
Sauerkraut	3	3.4	1.8–5.4
Choy			
Bok choy/Pak choy	3	4.3	1.7–6.5
White Baby Bok Choy	3	4.7	2.5–6.5
Green Baby Bok Choy	3	5.6	2.5–9.3
Yu Choy	3	2.1	1.0–3.5
Greens			
Collard greens	3	0.7	0.3–1.2
Kale	5	2.6	0.3–10.3
Mustard greens	3	178.9	101.7–331.9
Turnip greens	3	4.3	3.4–5.8
Roots			
Daikon	3	57.6	29.6–95.2
Kohlrabi	3	5.3	2.5–8.9
Radish	4	6.4	5.8–7.4
Turnip	3	31.4	22.4–47.4
Condiments			
Yellow Mustard	3	6.4	1.2–16.4
Mayonnaise	3	2.8	2.1–3.3
Horseradish	4	295.1	87.1–639.8
Wasabi	3	211.4	178.3–237.9

### 3.2 Effect of cooking methods on ITC yields

**Figure 2:**
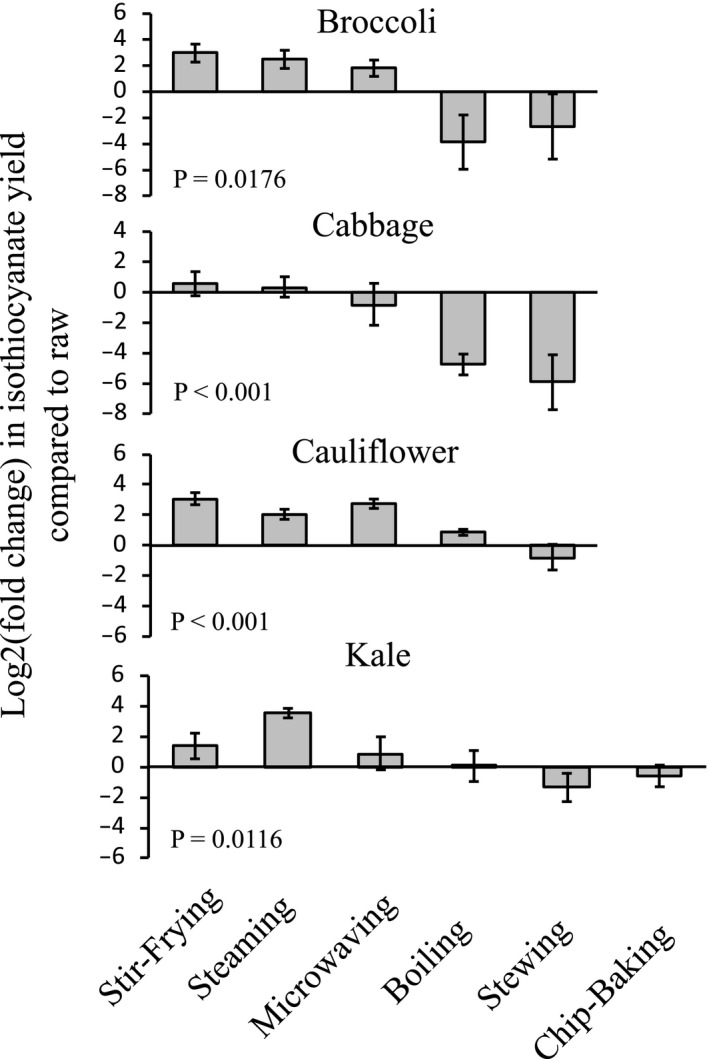
Effect of cooking methods on total isothiocyanate yield from four commonly consumed cruciferous vegetables. At least three samples per vegetable were tested. Fold changes in total isothiocyanate yields between cooked and raw counterparts were calculated and compared using one‐way ANOVA (analysis of variance) after log2 transformation. Values are mean ± *SEM*

To understand the effect of cooking methods on ITC yields, four commonly consumed cruciferous vegetables (broccoli, cabbage, cauliflower, and kale) were cooked under conditions representing stir‐frying, steaming, microwaving, boiling, stewing, and chip‐baking (for kale only). The ITC yields under different cooking conditions are presented in Table 3, along with fold changes of the yields over their raw counterparts. Fold changes were log2 transformed and compared among cooking methods. As shown in Figure 2, cooking significantly altered the yields of ITCs from all four vegetables (*p* < .05), although the changes were at various levels in response to each cooking method. For example, stir‐frying increased the ITC yield by almost 11‐fold for broccoli and eight‐fold for cauliflower, but only 2.8‐fold for kale and 2.3‐fold for cabbage; while stewing decreased ITC yields by 99%, 60%, 54%, and 50% for cabbage, kale, broccoli, and cauliflower, respectively (Table 3). Among the four vegetables, cabbage was more subjected to loss of ITC production after cooking, as both boiling and stewing reduced the ITC yield substantially, but stir‐frying, steaming, and microwaving had either no impact or caused slightly increases in ITC yield in comparison with raw. In contrast, cauliflower had substantial increases in ITC yield by stir‐frying, steaming, and microwaving but was less affected by boiling and stewing (Figure 2). In general, stir‐frying, steaming, and microwaving increased ITC yields, whereas boiling, stewing, and chip‐baking decreased ITC yields, in comparison with the raw counterparts.

**Table 3:** The yield of isothiocyanates from four major cruciferous vegetables under different cooking methods

	*N*	ITC µmol/100 g Mean (range)	Fold change Median (IQR)
Broccoli			
Raw	4	5.7 (2.7–11.7)	1
Stir‐fried	4	34.2 (4.8–52.8)	10.9 (5.5)
Steamed	4	40.1 (9.4–93.5)	6.1 (6.6)
Microwaved	4	28.8 (3.0–53.7)	3.9 (3.0)
Boiled	4	1.3 (0–2.6)	0.26 (0.4)
Stewed	4	5.6 (0–15.5)	0.46 (0.8)
Green/white cabbage			
Raw	4	57.6 (40.2–67.1)	1
Stir‐fried	4	132.2 (23.6–244.3)	2.3 (2.8)
Steamed	4	100.7 (13.1–167.5)	1.7 (0.7)
Microwaved	4	66.4 (2.3–136.0)	1.2 (0.7)
Boiled	4	2.9 (0.7–6.1)	0.04 (0.04)
Stewed	4	4.7 (0–16.9)	0.01 (0.1)
Cauliflower			
Raw	4	2.3 (1.3–2.9)	1
Stir‐fried	4	19.2 (14.4–24.2)	7.9 (5.6)
Steamed	4	9.5 (7.2–14.1)	4.4 (2.9)
Microwaved	4	14.9 (13.0–17.0)	6.5 (3.4)
Boiled	4	4.3 (2.4–6.6)	1.8 (0.3)
Stewed	4	1.7 (0.5–4.1)	0.5 (0.9)
Kale			
Raw	5	2.7 (0.3–10.3)	1
Stir‐fried	5	3.8 (0.6–10.6)	2.8 (7.0)
Steamed	5	24.1 (3–91.6)	8.8 (4.5)
Microwaved	5	10.5 (0.3–44.7)	1.3 (3.5)
Boiled	5	1.3 (0.3–2.3)	0.8 (3.6)
Stewed	5	0.3 (0.1–0.5)	0.4 (0.6)
Chip‐baked	4	0.4 (0.3–0.6)	0.8 (0.6)

### 3.3 Composition of weighting factors for estimation of ITC yields from cooked vegetables

**Figure 3:**
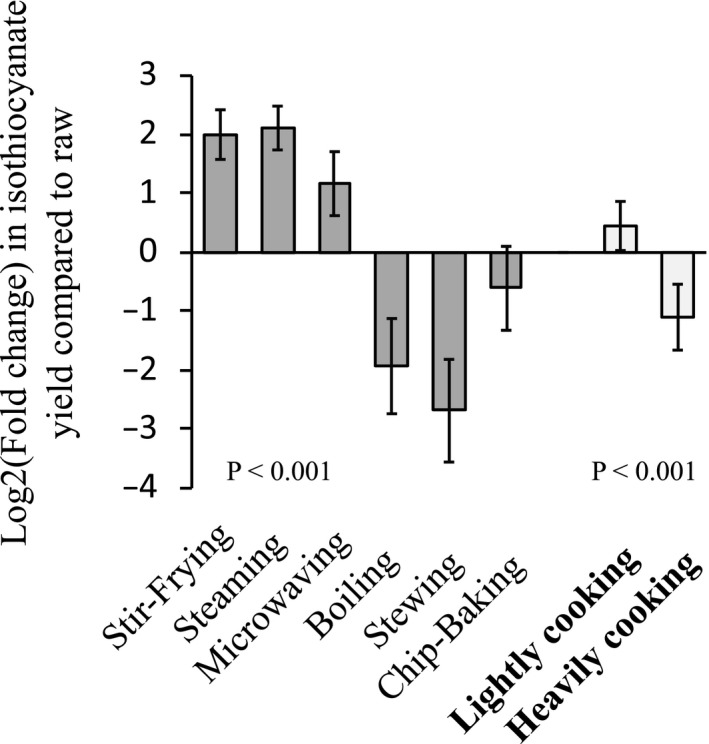
Summary of cooking effect on total isothiocyanate yield. Lightly cooking included stir‐frying, steaming, and microwaving. Heavily cooking included boiling, stewing, and chip‐baking. Fold changes in total isothiocyanate yields between cooked and raw counterparts were log2 transformed and subjected to one‐way ANOVA (analysis of variance). Values are mean ± *SEM*

The overall impact of cooking methods on ITC yields is summarized in Figure 3. Overall, cooking methods can be categorized into two groups based on the opposite effect on the ITC yield from the vegetables: lightly cooking, comprised of stir‐frying, steaming, and microwaving, increases the ITC yield by averagely 4.3‐fold; and heavily cooking, comprised of boiling, stewing, and chip‐baking, reduces the ITC yield by 58% in average. Both lightly cooking and heavily cooking significantly changed the ITC yield in comparison with raw (*p* <.05). The median fold change in ITC yield for each cooking method in comparison with raw is presented in Table 4. Stir‐frying, steaming, and microwaving increased the ITC yields by 5.0‐, 5.6‐, and 3.2‐fold, respectively; while boiling and stewing reduced the ITC yields by 60%, and a 20% decrease was observed by chip‐baking. These values can be considered as weighting factors to adjust the varied cooking effects on the ITC yield for estimation of ITC exposure from cruciferous vegetable consumption.

**Table 4:** The median fold changes in the yield of isothiocyanates from cruciferous vegetable under different cooking conditions in comparison with raw

	Fold change Median (IQR)
Stir‐frying	5.0 (7.7)
Steaming	5.6 (6.2)
Microwaving	3.2 (4.3)
Boiling	0.41 (1.7)
Stewing	0.42 (0.5)
Chip‐baking	0.80 (0.6)
Lightly cooking	4.3 (7.1)
Heavily cooking	0.42 (1.2)

## 4 DISCUSSION

In this study, we compiled a comprehensive list of ITC‐containing foods including 21 types of cruciferous vegetables and four types of condiments and surveyed the ITC yield under the raw condition. A wide range of ITC yields was observed among vegetables with an over 295‐fold difference between the lowest (0.7 μmol/100 g wet weight in collard greens) and the highest yield (206.9 μmol/100 g wet weight in arugula). There was no general pattern in the ITC yields comparing roots and leafy cruciferous vegetables, although turnip roots had a higher ITC yield than its greens (31.4 vs. 4.3 μmol/100 g wet weight). The ITC yields were over 30 μmol/100 g wet weight for turnip and daikon, but low around 5–6 μmol/100 g wet weight for kohlrabi and radish, which is comparable to the yields from most leafy cruciferous vegetables. The large variations in ITC yields from cruciferous vegetables are consistent with findings from previous studies of nine types of cruciferous vegetables commonly consumed in Asia (Jiao et al., 1998), and eight types of cruciferous vegetables commonly consumed in the United States (Tang et al., 2013). Some condiments have long been hypothesized to contain ITCs. Our study is the first to document ITC levels in condiments. Indeed, a considerable amount of ITCs was found in four tested condiments, and horseradish had the highest level (295.1 μmol/100 g wet weight) among all tested food items. Overall, across all 25 food items tested in the study, ITC yields varied over 420‐fold. These findings further emphasize the importance of comprehensive composition data to estimate and understand ITC exposure from human diet.

Not surprisingly, variations in ITC yields also exist within each type of vegetable as well as condiment, ranging from 2.8 μmol/100 g to 295.1 μmol/100 g across samples. Various factors may contribute to this intravariation, including rainfall and fertilization for planted vegetables, genetic variations of vegetable strains, and storage and transportation for harvested vegetables, which all play roles in determining the content of glucosinolates in vegetables (Verkerk et al., 2009). For example, different sulfur and nitrogen soil content during fertilization caused significant differences in the amount of glucosinolates in vegetables (Mithen et al., 2003). Concentrations of glucosinolates also vary in vegetables with different genetic backgrounds, even vegetables grown under uniform cultural conditions (Kushad et al., 1999). Furthermore, harvest time also seems to affect the content of glucosinolates (Verkerk et al., 2009). Fahey et al. reported that the glucosinolate content in mature broccoli is about 15 times lower than it is in three‐day‐old broccoli sprouts (Fahey, Zhang, & Talalay, 1997). Indeed, a five‐fold higher ITC yield was found from broccoli sprouts than mature broccoli in our study. Refrigerated storage and transportation also led to a loss in glucosinolates (Vallejo, Tomás‐Barberán, & García‐Viguera, 2003). Compared to an over 420‐fold difference in ITC yields across all types of vegetables and condiments, the level of intravariation is less with a maximum of 30‐fold difference within each type of sample. In addition, previous studies also showed different measurements from vegetables purchased at different time points, suggesting that season may also contribute to the variations in the ITC yield (Tang et al., 2013). Overall, the intravariations in ITC yields add a certain degree of uncertainty in estimation of ITC exposure from human diet. This uncertainty is further compounded by vegetable cooking methods.

The varied effects of cooking methods on ITC yields are striking. Using the ITC yield from the raw cruciferous vegetable as the reference, we found that boiling, stewing, and chip‐baking reduced ITC yields; whereas stir‐frying, steaming, and microwaving increased ITC yields from cruciferous vegetables. The patterns are similar among all four vegetables (Figure 2). The extent of change was not trivial, showing a maximum 11‐fold increase by stir‐frying in broccoli and a 99% reduction by stewing in cabbage. The opposite effect of cooking methods on ITC yields could be explained by factors coexisting with glucosinolates in vegetables, in particular, myrosinase, and ESP. Unlike in condiments, ITCs in vegetables are stored as glucosinolates and are released upon plant cell disruption during vegetable chopping, chewing, or cooking. The hydrolysis of glucosinolates is initiated by myrosinase, but ESP shifts the reaction toward the production of nitriles at the cost of ITCs as discussed in Introduction. Interestingly, ESP seems more heat‐labile than myrosinase. Matusheski et al. showed that heating at 60–70°C for 5–10 min destroys ESP but spares myrosinase, resulting in an increases of ITC yields by approximately three‐fold to seven‐fold, but further temperature increase caused declines of ITC yields, probably due to inactivation of myrosinase (Matusheski et al., 2004; Sosińska & Obiedziński, 2011). The differential impact of heating on myrosinase and ESP correlates well with our findings that the ITC yields are higher in lightly cooked vegetables but lower in heavily cooked vegetables, in comparison with raw counterparts that theoretically have the highest myrosinase activity without involvement of heating. Therefore, the ITC yield from cruciferous vegetables is not totally determined by myrosinase activity in the vegetable, but rather by the ratio of myrosinase and ESP spared under different cooking conditions. In our study, although all vegetables were cooked in the oven at the same temperature, heat penetration would be varied due to vegetable structure, cooking time, and amount of water added under each cooking method, which may explain the observed variability in ITC yields across four vegetables cooked under the same conditions (Figure 2). Besides affecting hydrolysis efficiency, cooking also changes the amount of available glucosinolates. Previous studies showed that cooking with water reduced glucosinolate content in cruciferous vegetables (Gliszczyńska‐Świgło et al., 2006; Rosa & Heaney, 1993; Vallejo, Tomás‐Barberán, & Garcia‐Viguera, 2002), probably due to leaching into the cooking water as glucosinolates are water‐soluble. To note, there were no detectable ITCs in the cooking water from boiling, stewing, and steaming in our study (results not shown). Interestingly, microwaving and steaming were reported to increase the level of glucosinolates (Gliszczyńska‐Świgło et al., 2006; Lu, Pang, & Yang, 2020), which was assumed that mild‐heating causes disintegration of plant tissues, thus leads to more release of glucosinolates (Nugrahedi, Verkerk, Widianarko, & Dekker, 2015). However, the potential contribution of heating‐caused inactivation of myrosinase to the increase of unhydrolyzed glucosinolates could not be excluded. It should be noted that the stir‐frying and microwaving in this study were all conducted for a relatively short time. Long‐term cooking even without water could also result in reduction of ITC yield, which is supported by the low yield of ITCs from chip‐baked kale in the study.

Two limitations should be considered. First, vegetable cooking was conducted in the laboratory using conditions mimicking different cooking methods, which might not truly represent the “real kitchen” cooking and thus introduce variations in evaluating cooking effects on ITC yields. Before the formal implementation of cooking conditions in the laboratory, several rounds of cooking experiments in the laboratory and in the real kitchen were conducted to optimize the best conditions mimicking real cooking in the kitchen. After the conditions were set up, a large batch of broccoli was purchased, chopped, mixed, and aliquoted randomly to complete all six cooking conditions in parallel in the laboratory and in the kitchen with the same ratio of added water for the same amount of cooking time. The ITC yields differed to some extent depending on cooking conditions between the samples cooked in the laboratory and in the kitchen, but the trends were consistent, showing higher yields from stir‐frying, microwaving and steaming, and lower yields from boiling, stewing, and chip‐baking in comparison with the raw samples (results not shown). Therefore, we are confident that the cooking conditions used in the study capture the general direction and extent of changes in ITC yields under different cooking methods. This serves the study purpose to provide generalized weighting factors in consideration of various cooking methods for estimation of ITC exposure from dietary intake of cruciferous vegetables.

Second, total ITC levels were measured instead of yield of individual ITCs from cruciferous vegetables. Individual ITCs differ by side‐chain structure and have shown variations in anti‐cancer potencies via different targets and/or mechanisms (Jiao et al., 1994; Navarro, Li, & Lampe, 2011; Singh & Singh, 2012; Wang et al., 2011). However, the characteristic structure of –N=C=S for all ITCs is primarily responsible for the bioactivity of ITCs by interacting with cellular targets. Therefore, ITCs collectively share a certain degree of cancer chemopreventive and/or therapeutic potential, making it important to capture total ITC exposure from diet for evaluation of its role in cancer. For individual ITCs, the yield could be derived proportionally by leveraging glucosinolate composition data in each cruciferous vegetable (Agudo et al., 2008; Kushad et al., 1999; McNaughton & Marks, 2003; Verkerk et al., 2009).

Our goal was to provide evidence‐based cooking recommendations for the general population to increase ITC exposure from consumption of cruciferous vegetables and give tools for researchers to estimate dietary ITC intake based on food consumption and cooking methods. To convey the information effectively, we summarized cooking methods into two categories and calculated weighting factors to consider the effect of cooking on ITC yields. Lightly cooking including stir‐frying, steaming, and microwaving caused an averagely four‐fold increase in ITC yields. In contrast, heavily cooking, comprised of boiling, stewing, and chip‐baking, led to a reduction of ITC yields by an average of 58%. However, the composition data and weighting factors provided in the study are by no means aimed for an accurate estimation of dietary ITC exposure. Not only the intravariation in ITC yields within each type of vegetables and variability in response to cooking methods, *in vivo* hydrolysis of ingested glucosinolates by microflora in the gastrointestinal tract (Kensler et al., 2005) also adds uncertainty in estimation of ITC exposure. To accurately capture ITC exposure, internal biomarkers, such as urinary ITC level or ITC‐albumin adduct, should be considered, although the internal biomarkers are more likely to reflect recent exposures. Based on food frequency data collected in epidemiologic studies, composition data and weighting factors could help understand long‐term exposure as well as exposure levels in general populations.

## 5 CONCLUSION

In conclusion, the current study surveyed ITC yields from a comprehensive list of food items commonly consumed in Western diet and assessed effects of various cooking methods on ITC yields from cruciferous vegetables. Food composition data were provided for 21 types of cruciferous vegetables and four condiments. Weighting factors were calculated for different cooking methods including lightly cooking (stir‐frying, steaming, and microwaving) and heavily cooking (boiling, stewing, and chip‐baking) separately for adjustment of cooking effect on ITC yields. Given the growing interest in dietary ITCs due to their cancer‐prevention potential, the data provided in this study will help researchers better estimate dietary ITC exposure to determine the relationship with human diseases in epidemiological studies, but also guide general populations on dietary ITC intake by making more informed choices for cooking and consumption of vegetables.

## CONFLICTS OF INTEREST

The authors report no conflict of interest.

## AUTHOR CONTRIBUTIONS

L. Tang, M. Kwan, J. Roh, L. Kushi, K. Danforth, Y. Zhang, and C. Ambrosone conceptualized and designed the research. Z. Wang and R. Pratt performed the research. Z. Wang and L. Tang analyzed the data and wrote the manuscript. M. Kwan, J. Roh, L. Kushi, K. Danforth, Y. Zhang, and C. Ambrosone critically reviewed the manuscript. All authors commented, read and approved the final manuscript.

## ETHICAL APPROVAL

This study does not involve any human or animal testing.
